# Seed production areas for the global restoration challenge

**DOI:** 10.1002/ece3.2455

**Published:** 2016-09-28

**Authors:** Paul G. Nevill, Sean Tomlinson, Carole P. Elliott, Erin K. Espeland, Kingsley W. Dixon, David J. Merritt

**Affiliations:** ^1^ Kings Park and Botanic Garden Kings Park WA Australia; ^2^ School of Plant Biology University of Western Australia Nedlands WA Australia; ^3^ School of Veterinary and Life Sciences Environment and Conservation Sciences Murdoch University Murdoch WA Australia; ^4^ USDA‐ARS Pest Management Research Unit Sidney MT USA; ^5^ Present address: Department of Environment and Agriculture ARC Centre for Mine Restoration Curtin University Bentley 6102 WA Australia

## Abstract

Wild‐collected seed can no longer meet global demand in restoration. Dedicated Seed Production Areas (SPA) for restoration are needed and these require application of ecological, economic, and population‐genetic science. SPA design and construction must embrace the ecological sustainability principles of restoration.

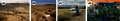

## 1 Introduction

Ecological restoration is the process of assisting the recovery of an ecosystem that has been degraded, damaged, or destroyed (SER, 2004). The achievement of global terrestrial restoration targets requires the large‐scale reintroduction of plants of wild species and regardless of whether large‐scale plant‐reintroductions are to be achieved through planting seedlings, or through the direct sowing of seeds to site, the availability and effective use of seeds is fundamental to success (Kettle et al., 2011; Leger & Baughman, 2015; Merritt & Dixon, 2011). However, shortfalls in seed supply for restoration are well recognized (Broadhurst, Driver, et al., 2015; Broadhurst, Hopley, Li, & Begley, 2015; Merritt & Dixon, 2011; Smith, 2014; Wijdeven & Kuzee, 2000) and the current and future demand for seeds far exceeds the volume that can be practically, economically, and ethically sourced from the wild (Broadhurst, Driver, et al., 2015; Broadhurst, Hopley, et al., 2015; Tischew, Youtie, Kirmer, & Shaw, 2011). Landscape‐scale restoration is underway across the globe in tropical, temperate, and dryland areas (Gritzner, Milan, & Berry, 2011) and ambitious restoration targets are recognized as necessary to arrest a host of negative environmental and social factors caused by land degradation. The global assessment of forest genetic resources adopted by the FAO in June 2013 (FAO, 2013) calls for policymakers to reinforce national seed programs to provide sufficient quantities of genetically appropriate seeds (see below for definition) for plantations and restoration, acknowledging a recently updated IUCN commitment to restore an astonishing 3.5 million square kilometers globally by 2030 (UNEP & IUCN, 2014). In many regions of the world, restoration relies largely, if not entirely, on sourcing seeds from wild plant populations (Broadhurst et al., 2015; Broadhurst, Hopley, Li, & Begley, 2015; Kettle et al., 2011), particularly where local provenance seed is desired (Hufford & Mazer, 2003). However, wild plants can be difficult to find and access or have sporadic and unpredictable seed production from year to year (Borders, Cypher, Ritter, & Kelly, 2011; Kettle, 2010). Furthermore, land clearing activities and changed land‐use patterns have fragmented vegetation and reduced the extent from which seeds can be sourced (Boshier et al., 2015; Walker et al., 2004). We suggest that seed production areas (SPA) are central to overcoming shortfalls in seed availability that prevent effective and timely restoration of plant communities. Ambitious global restoration goals and the associated need to restore a greater variety of ecological systems (e.g., grasslands, deserts, forests, woodlands) mean that the implementation of large‐scale seed production programs as acknowledged in the global policy and science sectors must be progressed for diverse wild plant species. Appropriately developed SPA will alleviate the potential for overharvesting of wild plant populations and improve the quantity and reliability of seed available (Broadhurst et al., 2008; Broadhurst, Hopley, et al. 2015). For example, 28,000 ha of remnant North American tall grass prairies are targeted for restoration (Gerla, Cornett, Ekstein, & Ahlering, 2012); however, these native grasslands currently occupy between 0.01% and 2.5% of their historical range (Packard & Mutel, 2005). In such situations, harvesting of seeds from wild plants has both ethical and practical constraints on restoration pace and success because so few seed sources remain. The constraint is particularly strong when local genetic material is required or encouraged (EPA, 2011; Mijnsbrugge, Bischoff, & Smith, 2010).

**Figure 1:**
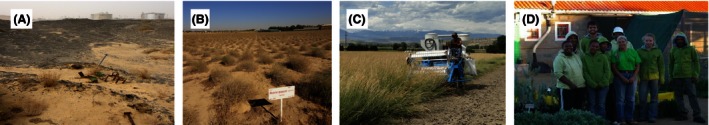
The Iraqi invasion of Kuwait left almost 10% of the desert areas (A) denuded when oil wells were ignited and much of the vegetation cover was destroyed. This scale of restoration will require intensive seed farming with trial sites now underway (B) (Photographs by Kingsley Dixon). Up until now, seed farming has focused on wind‐pollinated, grassland/rangeland species, but we need to understand how to produce seeds effectively for a variety recipient ecological systems (C) (Harvesting of seed Montana USA: Photograph by Joe Scianna). Engagement in seed production areas for restoration of grazing damaged and postmined areas in Karoo rangeland, South Africa, has given traditional communities economic empowerment and independence (D) (Photograph by Sue Milton‐Dean)

Seed production areas are purpose‐designed for seed production and can be established as monocultures or species mixtures. They exist at many scales, from small‐sized, intensively managed seed beds within onsite nurseries or contracted commercial growers (Gibson‐Roy, Moore, Delpratt, & Gardner, 2010; Koch, 2007), to large‐scale seed exchange networks and government‐coordinated programs (Haslgrübler et al., 2011; Shaw, Pellant, Fisk, & Denney, 2012). SPA are designed for different purposes which includes the production of improved germplasm with desirable traits such as increased vigor and/or disease resistance, and the production of germplasm capturing and retaining local or regional genetic diversity. There are highly developed, sophisticated native seed industries for forestry and forage grasses in North America (Chivers, Jones, Broadhurst, Mott, & Larson, 2016; Johnson et al., 2010; Oldfield & Olwell, 2015) and Europe (Haslgrübler, 2016); these must be extended and designed for a greater diversity of species and ecosystems to meet global demand (Fig. 1).

**Figure 2:**
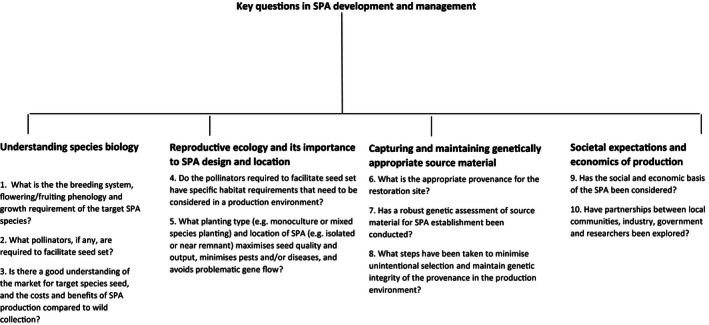
Key questions that must be addressed in the development of seed production areas

**Figure 3:**
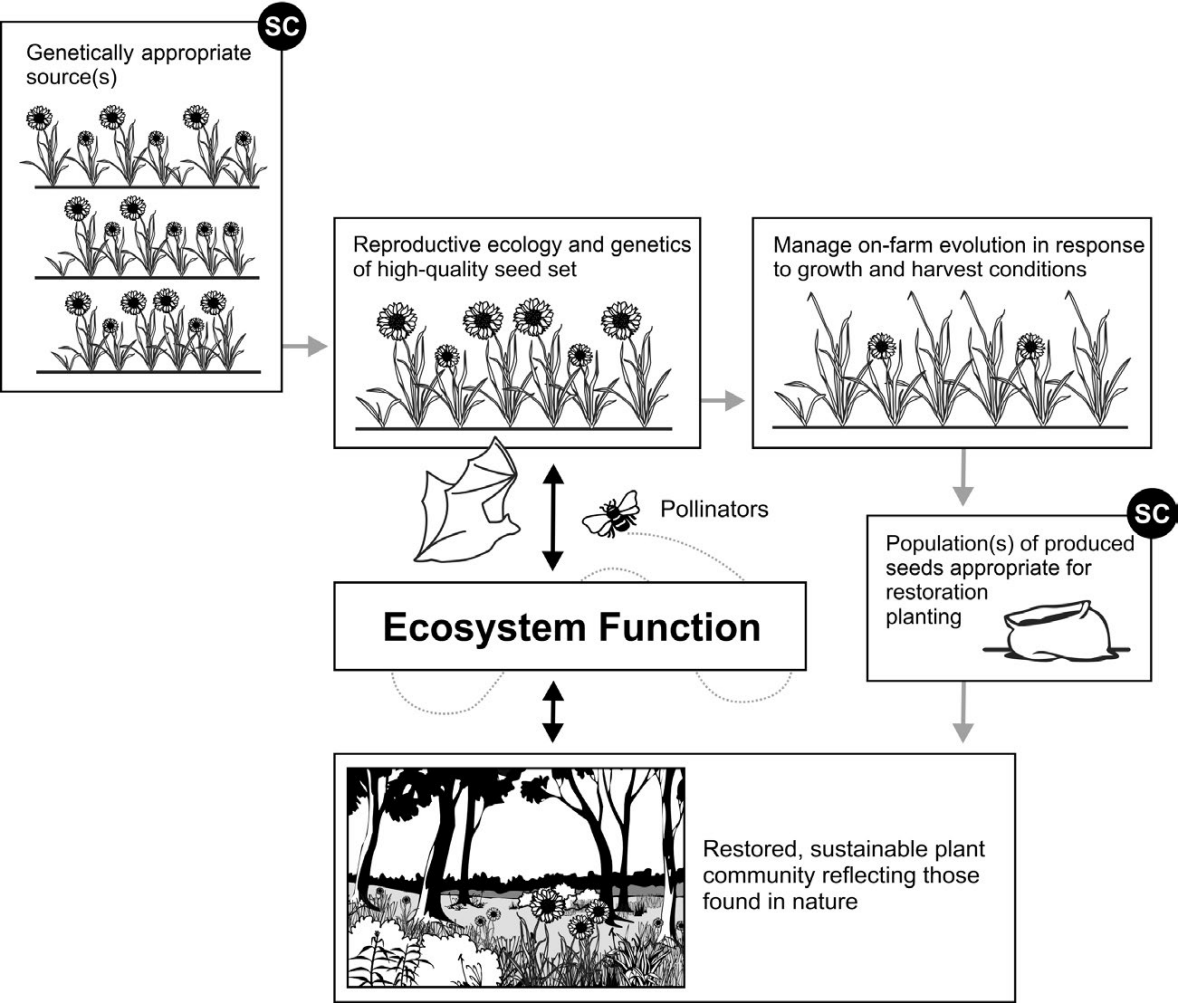
Relationships of biology, ecology, and genetics of seed production areas (SPA) and interrelated ecosystem function. Enforceable seed certification (SC) provides quality assurances for collectors, producers, and end‐users, promoting appropriate genetic management practices and ecologically and economically sustainable SPA

However, we caution against focusing solely on maximizing seed production, as this may be a source of both intentional and unintentional selection that increases agronomic/domestication trait frequencies in populations (Basey, Fant, & Kramer, 2015; Espeland et al., 2016). Many traits selected for in typical seed production environments (such as seed shattering, low dormancy, and synchronous phenology) are maladaptive in the wild (Espeland et al., 2016). Extreme cases of this type of selection are native plant cultivars or other improved varieties. Not only are improved varieties often phenotypically invariant (Espeland & Hammond, 2013; Leger & Baughman, 2015) and therefore unlikely to respond to selection imposed by climate change and other adaptive hurdles (Espeland et al., 2016), but they have often been developed specifically for traits such as above‐ground biomass accumulation, herbicide tolerance, or suitability for mechanized harvesting (Chivers, et al. 2016 and references therein) that may be maladaptive in the long term in some restoration environments (Leger & Baughman, 2015). Although cultivars and non‐native species might be considered the most cost‐effective and readily available seed varieties when short‐term goals like soil stabilization cannot be achieved with native accessions (D'Antonio & Meyerson, 2002; Jones, Monaco, & Rigby, 2015), they cannot be considered a cost‐effective choice when the goal of restoration is to sustain diverse native landscapes and the native wildlife that depend on them (Kuebbing & Nuñez, 2015). There is little evidence that successful cultivar or non‐native plantings become desirable native plant communities (D'Antonio & Meyerson, 2002; Kettenring, Mercer, Reinhardt Adams, & Hines, 2014; Prach & Hobbs, 2008). Therefore, we advocate for SPA to embrace the guiding principles of restoration (SER, 2004), producing seeds that create self‐sustaining plant communities which are resilient to future disturbances. Here we highlight 10 key questions that must be addressed in the development of SPA (Fig. 2) and outline a systematic approach for integrating species biology, reproductive ecology, ecosystem function, genetic integrity, and economic sustainability to meet current and future needs for restoration propagules (summarized in Fig. 3).

## 2 Key Factors that must be Considered in the Development of SPA

Effective SPA requires an understanding of ecosystem processes (e.g., pollination) and entails coming to terms with the biotic (e.g., plant and animal interactions) and abiotic (e.g., temperature, rainfall, and exposure) conditions underpinning productivity and genotype. Producing seed for restoration requires the understanding and/or management of four key factors (discussed below):

1. Understanding species biology;
2. Reproductive ecology and the influence of SPA location and design;
3. Capturing and maintaining genetically appropriate source material; and
4. Social expectations and economics of production.

The choice of what species to include in SPA, and the location and design of SPA, cannot be one‐size‐fits‐all and must be based on species biology and landscape context. Species‐level decisions will depend on the importance of obtaining seed for a particular species, the difficulty in producing that seed in a SPA, and the likelihood of obtaining that seed from the wild. Basic biological knowledge is required for these decisions to be made in an informative manner. Breeding systems, flowering phenology, pollinator dependence, water, light and nutrient requirements, and pest and disease control all need to be considered in order to produce viable seeds. It must be acknowledged that some life‐history traits (such as r‐strategic perennial grasses) lend themselves more readily to rapid and high volume production and that it is easier to develop SPA for such species than others (such as k‐strategic woody perennials). Also, the production context, ranging from monoculture to diverse mixed species plantings, will be dependent on such species characteristics. For example, species that are ideal for growth in isolated monocultures include those that are wind pollinated or pollinated by nonspecific vertebrates with broad habitat requirements. In contrast, SPA for the restoration of ecosystems will often involve woody perennials that mature slowly, take up a large amount of space, and potentially require the support of complex ecosystems (such as pollinator networks). Plants with specialized pollinator requirements include those with arthropod mutualisms (e.g., buzz pollinators; Gaskett, 2011) as well as partnerships with birds and mammals (Fleming & Muchhala, 2008). When plants with specific mutualisms are critical for restoration, pollinator communities may also need to be restored or optimized in SPA, potentially requiring a diverse plant species composition to support more of the needs of the pollinator (e.g., nesting habitat). While mixed plantings will be more difficult to harvest and manage than a monoculture, structuring diverse plantings in closely associated and alternating beds, can provide the integration between diverse floral resources required to perennially sustain pollinators, while maintaining the practicality of harvesting methods. Alternatively, SPA could be located near natural populations, thus requiring particular consideration of SPA placement in the landscape and an understanding of pollinator energetics and thermal biology to ensure securing their services (McCallum, McDougall, & Seymour, 2013; Menz et al., 2011; Tuff, Tuff, & Davies, 2016).

Due to the scale of operations that are required to meet restoration needs, and the variability inherent in managing a wild‐type crop, the internal structure of SPA will depend on both biology and ecology; therefore, best practices in the spatial arrangement of plants within SPA will be variable. Crop plants have been selected to produce high seed yields under conditions of high intraspecific densities (Weiner, Andersen, Wille, Griepentrog, & Olsen, 2010); however, native plants used for restoration may need more space in between plants (i.e., less intraspecific competition), or may need specific localized commensalisms (e.g., parasites or mycorhizae) in order to produce profitable yields (Felton et al., 2016). Increasingly, evidence suggests that field‐scale diversification promotes native pollination systems (Kremen & M'Gonigle, 2015), while high intraspecific densities within SPA may make populations more susceptible to disease (Dawson et al., 2012; Jensen, Dreiseitl, Sadiki, & Schoen, 2011; Lankau & Strauss, 2011; Shykoff & Bucheli, 1995). Disease management within SPA may require intercropping or even more careful attention to intraspecific genotypic diversity (Parker, 1986).

After the immediate biological and ecological practicalities of growing plants for seed production are addressed, consideration of genetic issues must take place. Locally adapted genotypes and/or maximized genetic diversity of restored populations are the foundation of resilient ecosystems (Broadhurst et al., 2008). The genetics of seed sourcing for restoration is a contentious issue (Boshier et al., 2015) and recommendations variously include, “local is best”, composite provenancing, admixture provenancing, climate‐adjusted provenancing, and predictive sourcing for climate change (Broadhurst et al., 2008; Havens et al., 2015; Prober et al., 2015; Williams, Nevill, & Krauss, 2014). For any chosen strategy, we suggest genetic management in plant material selection for SPA: (1) identify genetic boundaries at different taxonomic and spatial hierarchies (e.g., species, subspecies, and populations); (2) assess genetic diversity and ploidy levels within and among populations; and (3) evaluate adaptive traits (e.g., common garden trials or studies of molecular markers linked to adaptive variation). Recent advances in next‐generation sequencing methods have revolutionized the potential practical applications of genetics to restoration, greatly increasing capacity to assess adaptive genetic variation, monitor restored communities, and to measure the genetic resistance of restored populations (Williams, et al. 2014). This information will help delineate provenance zones where seed produced in SPA can be used, to guide the founding population size of the SPA, and ensure that the maximum amount of genetic diversity within a provenance is captured. Where provenance selection for a changing climate is a priority, seed from different provenances could be grown separately, and guided by the process described above, mixed at different ratios prior to use, depending on factors such as genetic risks and the rate and reliability of climate change predictions.

Robust genetic assessments that incorporate all three points above are rarely conducted for restoration. Identifying genetic boundaries and genetic diversity within and among species is common in threatened species conservation, but not other applications, and the evaluation of adaptive traits is widespread only in the western USA to determine regional seed transfer zones in wind‐pollinated tree and grass species (Bower, St Clair, & Erickson, 2014). Where funding is limited and/or SPA need to be developed rapidly, plant material for SPA establishment should be selected based on risk assessment protocols that assess genetic risk in revegetation (e.g., Byrne, Stone, & Millar, 2011). Following SPA establishment, active management is required to add genetic diversity and replace senescent plants in SPA to ensure an even mix of genotypes from each provenance source. In addition, the genetic quality of seeds produced in the SPA must be regularly monitored for adequate genetic diversity and evidence of inbreeding (Broadhurst, Driver, et al., 2015; Broadhurst, Hopley, et al. 2015).

Genetic management should also include the development of an enforceable seed certification system to provide assurances for end‐users, in the same way commercial agricultural and horticultural seed is subject to stringent certification protocols. It is crucial to define seed zones and develop quality assurance standards for all stages of the process (i.e., producing, selling, and using native seeds), thus requiring a certification system with mechanisms of control. In the USA, seed certification systems such as those under the Great Basin Restoration Initiative for the large‐scale production of forb seeds for rangeland management (Shaw, Lambert, DeBolt, & Pellant, 2004) have been in place for over a decade. In Western Australia, an accreditation system has recently been launched by the peak revegetation industry body to improve and standardize quality control for wild‐collected seeds (http://riawa.com.au/wordpress/?page_id=1059). The European Native Seed Science Technology and Conservation network, a newly formed consortium of academic institutions and commercial seed companies across Italy, the UK, and Netherlands, also has a project to develop certification of seed quality and provenance (http://nasstec.eu/forum/esr-11c). Certified seed and in particular regulations surrounding the number of generations permitted on‐farm before genetic replenishment is required would, for example, identify unwanted domestication of restoration taxa.

Once SPA are developed, there is a clear risk of unintentional selection in the production environment. Environmental effects on the plants producing seeds (i.e., maternal effects resulting from such factors as intraspecific density, irrigation, and fertilization) and vendor‐specific storage protocols may have lasting signatures on seed phenotypes (e.g., dormancy) that may promote or inhibit performance in restoration (Long et al., 2014). Risks of genetic erosion resulting from SPA may be minimized when the production context is ecologically similar (e.g., plant density, moisture, light, or disturbance levels) to the wild collection location(s) and/or the ultimate restoration planting environment (Espeland et al., 2016). This approach may reduce seed yields in the short term, but will help ensure long‐term genetic fitness (Kettenring et al., 2014). Government‐run plant material centers in the USA control for on‐farm response to selection (e.g., Dawson et al., 2012) by replanting their grass and forb production fields every few years. Preserving genetic diversity of carefully provenanced populations in SPA may be challenging, particularly for long‐lived species or those in polyculture. However, the costs of failed restoration due to inappropriate seed choices are rarely accounted for (Zedler, 2007), and economic incentives for producing sustainable seed must be considered from a policy standpoint.

While ecological factors are critical to successful SPA, the social and economic basis of SPA enterprises cannot be ignored. The priorities we describe so far for the promotion of diverse seed for restoration outcomes are the familiar objectives of a scientific and conservation community that strives for the return of biodiverse, functional, and resilient ecosystems to disturbed landscapes (e.g., Bozzano et al., 2014; McDonald, Jonson, & Dixon, 2016; Plant Conservation Alliance 2015). These priorities, however, are dramatically juxtaposed with the economic priorities of agronomy. This often leads to conflict between the aims of the scientific community and the seed production industry. Reconciling these conflicting views is one of the most difficult and important issues to address in the development of SPA. While restoration ecologists commonly advocate genetically diverse, provenance‐specific products (Bozzano et al., 2014), the seed industry maintains such an approach which leads to a product of such high cost to produce, or such specialist need, that market forces do not justify its production. In order to profit, the seed industry commonly advocates artificial selection as a means to develop rapidly maturing seed crops amenable to cultivation for species that can be sold across a large geographic region (Chivers, Jones, Broadhurst, Mott, & Larson, 2016). However, if these products do not meet restoration goals, then their value should be very low. Identifying intermediate seed transfer zones that are large enough to be profitable to the seed industry but small enough to provide locally adapted seed for restoration would be a solution for suitable species. Additionally, capital investment by the mainly governmental agencies responsible for land management, or taxation concession in recognition of the specific costs and critical ecological value associated with species that take many years to reach reproductive maturity would undoubtedly accelerate establishment of SPA as a sustainable contribution to the green economy.

An additional challenge to the growth of not only SPA but restoration in general is the gap between restoration activities and the communities that surround them (Eitzel et al., 2012), particularly in developing countries (Brancalion, Viani, Aronson, Rodrigues, & Nave, 2012) where the area of degraded lands pledged for restoration is immense. While there are examples of successful community‐developed SPA (e.g., Renu‐Karoo South Africa; Milton‐Dean & Dean, 2015), these tend to be at a small scale. An alliance of traditional indigenous communities with business enterprises provides opportunities for access to business expertise while capitalizing on their intimate, traditional knowledge of local plant species. Multinational, resource‐intensive industries such as the mineral extraction sector have yet to develop large‐scale SPA, but there is a clear role for their engagement, potentially with the involvement of traditional communities. This is particularly so where, for example, in the northwest Australian Pilbara region, collective mining disturbance footprints are large (1,000 km^2)^ (EPA, 2014), and regulatory requirements for restoration following mining dictate the use of locally sourced seed (EPA, 2011). The infrastructural investment experience of the resource extraction sector could be easily turned to the industrial scaling of SPA and could be readily integrated into community outreach programs sponsored by such agencies (Solomon, Katz, & Lovel, 2008).

Given the scale of the global restoration targets, these types of partnerships between businesses, investors, governments, NGOs, and local people, perhaps initiated by organizations like the International Union for Conservation of Nature and their Business and Biodiversity program, are essential if SPA are to be successful. Not only do such organizations engage the business sectors that have a significant impact on natural resources, using various tools including mitigation of impacts, influencing policy and leveraging supply chains (Bishop, Kapila, Hicks, Mitchell, & Vorhies, 2008), they also promote communication between diverse stakeholders. Partnerships between the forestry industry, government, and researchers have been especially effective in defining propagation limitations for tree genetics in East Africa (e.g., Lengkeek et al., 2005) and are modeled organizationally by the Center for International Forestry Research (http://www.cgiar.org) that operates in Asia, Latin America, and Africa. Just like integration of local communities, economies, governments, and other stakeholders are required for restoration to be successful (e.g., Biswas, Mallik, Choudhury, & Nishat, 2009; Milton‐Dean & Dean, 2015; Walters, 1997), these collaborations are also required for successful SPA.

## 3 Conclusions

Achieving global restoration goals will be challenging and, as the diversity of habitats being restored grows, we need to understand how to produce seeds effectively for a variety of ecological systems. Providing a sound economic framework for appropriate seed production is essential, as is a science‐based approach for SPA establishment and maintenance that ensures that appropriately produced seeds contribute effectively to sustainable restorations.

## Funding Information

None declared.

## Conflict of Interest

None declared.

## Data Accessibility

This article does not contain new data.
